# Antiviral activity of 5-aminolevulinic acid against variants of severe acute respiratory syndrome coronavirus 2

**DOI:** 10.1186/s41182-021-00397-x

**Published:** 2022-01-07

**Authors:** Mya Myat Ngwe Tun, Takaya Sakura, Yasuteru Sakurai, Yohei Kurosaki, Daniel Ken Inaoka, Norifumi Shioda, Jiro Yasuda, Kiyoshi Kita, Kouichi Morita

**Affiliations:** 1 Department of Virology, Institute of Tropical Medicine (NEKKEN), Nagasaki University, 1-12-4 Sakamoto, Nagasaki, 852-8523 Japan; 2 Shionogi Global Infectious Diseases Division, Department of Molecular Infection Dynamics, Institute of Tropical Medicine (NEKKEN), Nagasaki University, Nagasaki, 852-8523 Japan; 3 Department of Emerging Infectious Diseases, Institute of Tropical Medicine (NEKKEN), Nagasaki University, Nagasaki, 852-8523 Japan; 4 National Research Center for the Control and Prevention of Infectious Diseases, Nagasaki University, Nagasaki, 852-8523 Japan; 5 Department of Genomic Neurology, Institute of Molecular Embryology and Genetics (IMEG), Kumamoto University, Kumamoto, Japan; 6 Graduate School of Pharmaceutical Sciences, Kumamoto University, Kumamoto, Japan; 7 School of Tropical Medicine and Global Health, Nagasaki University, Nagasaki, 852-8523 Japan; 8 Department of Host-Defense Biochemistry, Institute of Tropical Medicine (NEKKEN), Nagasaki University, Nagasaki, 852-8523 Japan

**Keywords:** SARS-CoV-2 variants, 5-ALA, SFC, Anti-viral drug

## Abstract

**Background:**

Genetic variants of severe acute respiratory syndrome coronavirus 2 (SARS-CoV-2) began to emerge in 2020 and have been spreading globally during the coronavirus disease 2019 (COVID-19) pandemic. Despite the presence of different COVID-19 vaccines, the discovery of effective antiviral therapeutics for the treatment of patients infected with SARS-CoV-2 are still urgently needed. A natural amino acid, 5-aminolevulinic acid (5-ALA), has exhibited both antiviral and anti-inflammatory activities. In a previous study, we demonstrated an in vitro antiviral effect of 5-ALA against SARS-CoV-2 infection without significant cytotoxicity. In the present study, we sought to investigate whether 5-ALA with or without sodium ferrous citrate (SFC) can inhibit in vitro both the original SARS-CoV-2 Wuhan strain and its variants, including the Alpha, Beta, Gamma and Delta strains.

**Methods:**

The antiviral activity of ALA with or without SFC was determined in Vero-E6 cell. The virus inhibition was quantified by real time RT-PCR.

**Results:**

Co-administration of 5-ALA and SFC inhibited the Wuhan, Alpha and Delta variants of SARS-CoV-2 with IC_50_ values of 235, 173 and 397 µM, respectively, and the Beta and Gamma variants with IC_50_ values of 1311 and 1516 µM.

**Conclusion:**

Our study suggests that 5-ALA with SFC warrants accelerated clinical evaluation as an antiviral drug candidate for treating patients infected with SARS-CoV-2 variants.

## Introduction

The outbreak of coronavirus disease (COVID-19) caused by severe acute respiratory syndrome coronavirus 2 (SARS-CoV-2) is a serious threat to global public health [1]. SARS-CoV-2 belongs to a beta-coronavirus subfamily and is a single-stranded positive RNA virus of roughly 29.9 kb in size [2]. Structurally, SARS-CoV-2 has a double-layered lipid envelope, including a spike glycoprotein (S), envelope protein (E), membrane protein (M) and nucleocapsid protein (N) [3]. The spike protein receptor-binding do main mediates recognition of the host cell receptor, angiotensin-converting enzyme 2 (ACE 2) [4, 5]. As of 18 October 2021, more than 241 million confirmed cases and 4.9 million deaths have been recorded in more than 220 countries around the world [6]. The clinical presentation of COVID-19 can vary from asymptomatic to mild, severe, or critical [7]; and serious respiratory system, gastrointestinal and neurological symptoms may arise as a result of infection [8–11].

Due to their low fidelity of genome replication, viruses acquire mutations over time, leading to variants of the original isolate. To date, thousands of genetic variants of SARS-CoV-2 are known to be circulating worldwide; however, four major SARS-CoV-2 variants of concern exist, namely, the Alpha (B.1.1.7), Beta (B.1.351), Gamma (P.1), and Delta (B.1.617.2) variants on Pango lineages [12]. Individual SARS-CoV-2 variants may display differences in transmission potential, virulence, clinical disease presentations (either milder or more severe), recognition by specific viral diagnostic tests, response by natural- or vaccine-induced immunity, and susceptibility to therapeutic agents [13–16]. Efficacious vaccines are being applied worldwide, and 39.3% of the world population has received at least one dose of a COVID-19 vaccine (i.e., 5.25 billion doses total) [17]. As of April 2021, 169 countries were actively administering vaccines, and the global vaccination campaign continues to progress [18]. Although a large fraction of the global population remains unvaccinated, the expectation of a continuously mutating virus that may come to exhibit at least partial resistance to vaccines emphasizes that drug development must proceed [19]. Moreover, it will take a long time to safely attain herd immunity against COVID-19 through vaccination, so effective therapies are required to prevent and treat COVID-19 [20].

Clinical investigations have focused on several approved antiviral drugs, including remdesivir [21], molnupiravir [22] and 3C-like protease inhibitors [23]. A natural amino acid, 5-aminolevulinic acid (5-ALA) commonly occurs in animals, plants, fungi and bacteria. So far, 5-ALA has been clinically used for metabolic improvement in human diseases, including diabetes [24] and the diagnosis and treatment of various cancers [25]. The conjugation of eight molecules of 5-ALA produces protoporphyrin IX (PPIX), which produces heme by the insertion of a ferrous ion [26]. Moreover, PPIX has shown antiviral effects against Dengue, Zika, chikungunya, and influenza A viruses [27–29]. The present study aims to evaluate the in vitro antiviral activity of 5-ALA against the Wuhan, Alpha, Beta, Gamma and Delta variants of SARS-CoV-2. Since previous papers on the potential of 5-ALA for heme oxigenas-1 induction [30] and anti-malarial activity [31] have shown that the addition of iron enhances the effect of 5-ALA, studies have been performed with and without iron. The ratio of 5-ALA to SFC was fixed as 4:1 according to our previous report on 5-ALA action to SARS-CoV-2 infection in Vero E6 and Caco-2 cells [32].

## Materials and methods

### Virus and cells

In this study, we used five SARS-CoV-2 strains; namely, the original Wuhan strain (hCoV-19/Japan/TY/WK-521,2019, GenBank_LC522975), the Alpha variant (hCoV-19/Japan/QK002/2020, EPI_ISL768526), the Beta variant (hCoV-19/Japan/TY8-612/2020, EPI_ISL1123289), the Gamma variant (hCoV-19/Japan/TY7-501/2020, EPI_ISL833366), and the Delta variant (hCoV-19/Japan/TY11-330-P1//2021, EPI_ISL2158613). The strains were provided by the Japan National Institute of Infectious Diseases and propagated in VeroE6 cells cultured in minimum essential medium supplemented with 10% fetal calf serum. Virus stocks were kept in a − 80 °C freezer as aliquots until testing. All experiments using infectious SARS-CoV-2 were performed in a biosafety level 3 (BSL3) laboratory at Nagasaki University according to standard BSL3 guidelines.

### Compounds

5-ALA was donated by Neopharma Japan (Tokyo, Japan) and was dissolved to 100 mM in water. Sodium ferrous citrate (SFC) was also donated by Neopharma Japan and was dissolved to 25 mM in water with 1 M of hydrogen chloride. Remdesivir (Gilead Sciences, Foster City, CA, USA) was dissolved to 10 mM in DMSO.

### Evaluation of antiviral activity assay

Remdesivir, 5-ALA-only, and 5-ALA with SFC were diluted at different concentrations in 2% fetal calf serum in minimum essential medium. For 72 h (h) before infection with SARS-CoV-2, Vero E6 cells were treated with the diluted compounds, then were seeded in 96-well plates. SARS-CoV-2, with a multiplicity of infection of 0.02, was subsequently added and incubated in the presence of the compounds at 37 °C until 48 h post infection (pi). Infected cell supernatants were then harvested and quantified by quantitative real-time reverse-transcription polymerase chain reaction (qRT-PCR). As ‘SFC only’ did not show antiviral activity in the previous report [24], the efficacy of ‘SFC only’ was not examined in the present study.

### Viral RNA extraction and quantitative real-time RT-PCR

A total of 100 μL of infected cell supernatant was harvested for viral RNA extraction by a Nextractor NX-48 robot, using an NX-48S viral Nucleic Acid (NA) kit (Genolution Inc., Seoul, South Korea) according to the manufacturer’s instructions. A total of 5 μL of RNA was used for quantitative real-time RT-PCR, and amplification of the N gene was performed using a total of 20 µL of reaction mixture consisting of 5 μL of Taqman master mix, 7 µL of nuclease water, 1 µL of 0.5-µM forward and reverse primers, and 1 μL of a 0.25-µM probe with SARS-CoV-2 N primers of the TaqMan Fast Virus 1-Step Master Mix (Life Technologies, Carlsbad, CA, USA) [33]. The primers and probes are referred to in more detail in our previous report [34].

### Cell viability assay

Vero E6 cells in 96-well plates were treated with samples for 3 days, and a cell viability assay was conducted in parallel with an antiviral assay. To determine the concentration that triggered a 50% reduction in cell survival (CC_50_), the results of the cell viability assay were evaluated by 3-(4,5-dimethylthiazol-2-yl)-2,5-diphenyl tetrazolium bromide according to the manufacturer’s instructions (Promega, Madison, WI, USA). Optical density was measured at 570 nm using a microplate reader (Synergy H1 M; BioTek Instruments, Winooski, VT, USA). Cell viability was determined using the following equation: cell viability (%) = (sample value)/(cell control) × 100. In addition, viral inhibition by 50% (IC_50_) was calculated as viral inhibition (%) = quantity of virus copies in (virus control − sample)/virus control × 100.

### Statistical analysis

Data were analyzed using GraphPad Prism version 9 (GraphPad Software, Inc., San Diego, CA, USA). Continuous variables are presented as mean ± standard deviation values. Group comparisons were performed using a one-way analysis of variance. A *t* test was used to compare continuous variables between two groups. For all calculations, a *P* value of less than 0.05 was considered to be statistically significant.

## Results

**Fig. 1:**
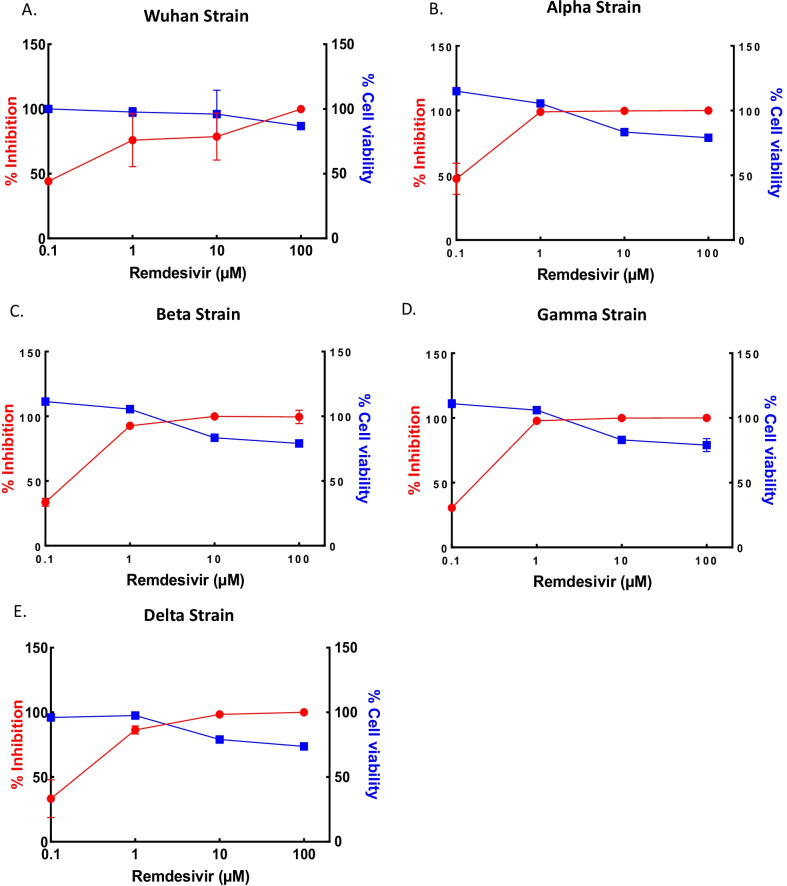
Corroboration of the antiviral effect of remdesivir against SARS-CoV-2 variants. Vero E6 cells were pretreated with remdesivir for 72 h and challenged with SARS-CoV-2. Infected cell supernatants at 48 h pi (MOI 0.02) were quantified by quantitative real time RT-PCR assay. The blue and red lines represent the CC_50_ and IC_50_, respectively; the blue squares represent cell viability (%) and the red circles represent SARS-CoV-2 infection inhibition (%). All experiments were performed in replicate

To evaluate the antiviral effects of the compounds, we first tested an approved antiviral drug, remdesivir, against the Wuhan, Alpha, Beta, Gamma and Delta SARS-CoV-2 strains; assayed as virus copy number in the cell supernatant using a specific qRT-PCR-based assay. The antiviral effect IC_50_ value of remdesivir was confirmed to be 0.1–1 µM against the Wuhan strain and four SARS-CoV-2 variants (Fig. 1).

**Fig. 2:**
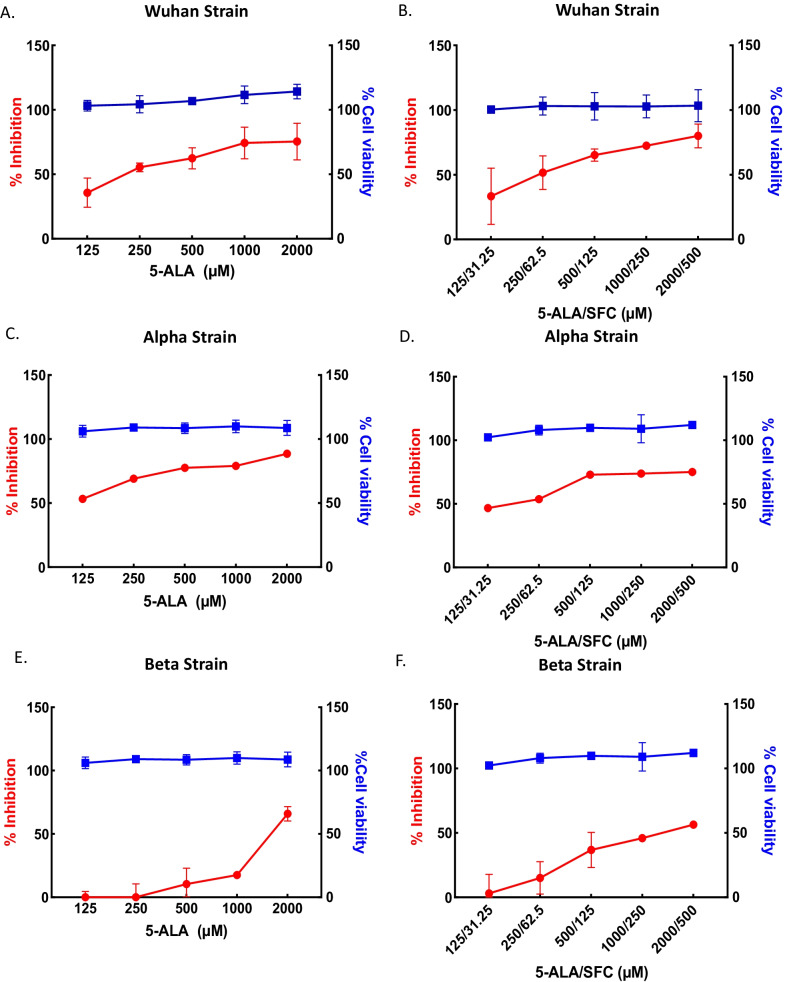
Antiviral effect of 5-ALA (panels **A**, **C**, and **E**) and 5ALA with SFC (panels **B**, **D**, and **F**) against Wuhan (panels **A** and **B**), Alpha (panels **C** and **D**) and Beta strains (panels **E** and **F**). Vero E6 cells were pretreated with ALA with and without SFC for 72 h and challenged with SARS-CoV-2. Infected cell supernatants at 48 h pi (MOI 0.02) were quantified by quantitative real time RT-PCR assay. The blue and red lines represent the CC_50_ and IC_50_, respectively; the blue squares represent cell viability (%) and the red circles represent SARS-CoV-2 infection inhibition (%). All experiments were performed in replicate

By applying the same qRT-PCR assay, the antiviral effects of 5-ALA and SFC were determined; and no cytotoxic effects with 2000 µM of 5-ALA and 500 µM of SFC in Vero E6 cells were observed. The resulting 5-ALA treatment and co-treatment with 5-ALA and SFC of VeroE6 cells inhibited the Wuhan strain of SARS-CoV-2 infection in a dose-dependent manner, with IC_50_ values of 207 and 235/59 µM, respectively (Fig. 2A, B). Furthermore, the antiviral effect IC_50_ values associated with 5-ALA and 5-ALA plus SFC treatments were 104 and 173/43 µM against the Alpha variant in a dose-dependent manner (Fig. 2C, D) and 1592, 1311/328 µM against the Beta variant in a dose-dependent manner (Fig. 2E, F), respectively.

**Fig. 3:**
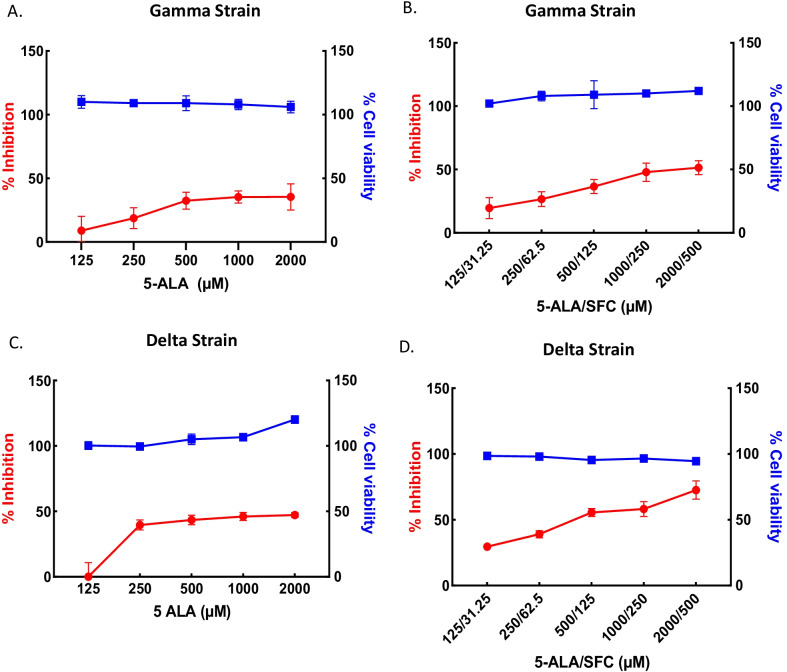
Antiviral effect of 5-ALA (panels **A** and **C**) and 5ALA with SFC (panels **B** and **D**) against Gamma (panels **A** and **B**) and Delta (panels **C** and **D**) strains. Vero E6 cells were pretreated with ALA with and without SFC for 72 h and challenged with SARS-CoV-2. Infected cell supernatants at 48 h pi (MOI 0.02) were quantified by quantitative real time RT-PCR assay. The blue and red lines represent the CC_50_ and IC_50_, respectively; the blue squares represent cell viability (%) and the red circles represent SARS-CoV-2 infection inhibition (%). All experiments were performed in replicate

Conversely, there was no viral inhibition and little viral inhibition after administration of 5-ALA to the Gamma (Fig. 3A) and Delta variants (Fig. 3C) SARS-CoV-2, respectively. However, co-treatment of 5-ALA and SFC led to viral inhibition IC_50_ values of 1516/379 and 397/99 µM after infection with the Gamma and Delta variants (Fig. 3B, D). The IC_50_ and CC_50_ of 5-ALA and 5-ALA with SFC against SARS-CoV-2 variants are described in Table 1.

**Table 1:** IC_50_ and CC_50_ values of 5-ALA and 5-ALA with SFC against SARS-CoV-2 variants

SARS-CoV-2 variants	Compound	IC_50_ (µM)	CC_50_ (µM)
Wuhan	5-ALA	207	> 2000
	5-ALA/SFC	235/58.7	> 2000/> 500
Alpha	5-ALA	104	> 2000
	5-ALA/SFC	173/43.2	> 2000/> 500
Beta	5-ALA	1592	> 2000
	5-ALA/SFC	1311/327.7	> 2000/> 500
Gamma	5-ALA	> 2000	> 2000
	5-ALA/SFC	1516/379	> 2000/> 500
Delta	5-ALA	> 2000	> 2000
	5-ALA/SFC	397/99.2	> 2000/> 500

In 5-ALA/SFC, the ratio of 5ALA to SFC was fixed as 4:1

*IC*_*50*_ 50% inhibition concentration, *CC*_*50*_ 50% cytotoxicity concentration, *5-ALA* 5-aminolevulinic acid, *SFC* sodium ferrous citrate

## Discussion

New clinical features due to multiple SARS-CoV-2 variants are occurring globally [35]. Despite the development of COVID-19 vaccines, the search for new therapeutics is still necessary. In our previous study, we investigated the antiviral effects of 5-ALA and SFC on SARS-CoV-2 clinical isolates from a Japanese patient using an immunofluorescence-based assay [32]. In this study, we report the virucidal activity of 5-ALA with and without concurrent SFC administration on the original strain of SARS-CoV-2 and several variants. A low concentration of 5-ALA with SFC could inhibit the Wuhan, Alpha and Delta strains, whereas a high concentration of 5-ALA with SFC acts against the Beta and Gamma strains. Thus, our results indicate that 5-ALA with SFC had antiviral effects against Wuhan and SARS-CoV-2 variants without cytotoxicity. Treatment with 5-ALA only inhibited the Wuhan, Alpha and Beta SARS-CoV-2 strains, suggesting a specific antiviral effect.

Recent research demonstrated that a molecular target, a G-quadruplex (G4) structure, is of potential interest given its antiviral activity, which inhibits SARS-CoV-2 replication [36, 37]. G4s are nucleic-acid secondary structures that may form in single-stranded DNA and RNA guanine-rich sequences under physiological conditions [38]. G4 structures have been reported in several DNA and RNA viruses, including coronaviruses, and implicated in the control of key viral processes [39]. Many algorithms have been developed for prediction of G4 propensity at the genome-wide level; namely, Quad-Parser, QGRS Mapper, G4P Calculator, QuadBase, cGcC score, G4Hunter, and G4RNA screener [40]. In the RNA genome of SARS-CoV-2, about 25 putative quadruplex forming sequences (PQSs) were found using QGRS Mapper [36, 41, 42], and G4RNA screener [43]. Among them, a G4 forming RNA in the coding sequence region of SARS-CoV-2 nucleocapsid phosphoprotein can be stabilized by a G4 ligand, a pyridostatin derivative. The expression of SARS-CoV-2 N were decreased both in vitro and in cells by a pyridostatin derivative [37]. Many G4 binding molecules have been reported as therapeutic strategies to target G4s, including porphyrins [43]. We previously reported that the intracellular production of the porphyrins PPIX and hemin, which are metabolized from 5-ALA, are novel G4 binding agents [44–47]. Here, we showed that exogenously supplied 5-ALA inhibited SARS-CoV-2 infection. This may be caused by increased generation of PPIX. These data indicate a novel therapeutic strategy that targets G4 against SARS-CoV-2 by intracellular generation of porphyrins from 5-ALA. However, the SARS-CoV-2 genome contains a significantly lower density of PQSs using the computational predicted algorithms, suggesting the G4 folding capacity of predicted SARS-CoV-2 PQSs consists of many ‘weak G4s’. As the PQSs do not differ between SARS-CoV-2 variants, other mechanisms of 5-ALA may be involved. Differences in the efficiency of virus invasion to the cell is one of the reasons. Since PQRs is only a bioinformatic analysis, it is necessary to identify G4s in the virus genome biologically in the future.

5-ALA is produced from most animals and plants, and thus us present in our food and may be taken by an oral route due to high bioavailability [48]. 5-ALA with SFC is a supplement formulation registered in Japan as a food with functional claims. In a recent clinical study, Japanese patients with COVID-19 who were given 5-ALA and SFC capsules orally experienced a shorter time to recovery than that reported for patients who received only standard care for SARS-CoV-2 infection [49]. Registration for the specified clinical trials on the effects of the treatment on human and its aftereffects has been completed and the data is now being analyzed (Japan Registry of Clinical Trials CRB 7180001 and 3190006, respectively).

## Conclusion

The present study demonstrates antiviral effects of 5-ALA and SFC against SARS-CoV-2 variants in vitro and may reveal these components as therapeutics and preventive measures for COVID-19.

## Data Availability

Not applicable.

## Abbreviations

SARS-CoV-2: Severe acute respiratory syndrome coronavirus 2
COVID-19: Coronavirus disease 2019
5-ALA: 5-Aminolevulinic acid
SFC: Sodium ferrous citrate
S: Spike glycoprotein
E: Envelope protein
M: Membrane protein
N: Nucleocapsid protein
ACE 2: Angiotensin-converting enzyme 2
PPIX: Protoporphyrin IX
BSL3: Biosafety level 3
qRT-PCR: Quantitative real time reverse-transcription polymerase chain reaction
NA: Nucleic acid
CC50: 50% Reduction in cell survival
IC50: 50% Virus inhibition
G4: G-quadruplex
PQSs: Putative quadruplex forming sequences
